# Brucellosis seroprevalence in Bali cattle with reproductive failure in South Sulawesi and Brucella abortus biovar 1 genotypes in the Eastern Indonesian archipelago

**DOI:** 10.1186/1746-6148-9-233

**Published:** 2013-11-26

**Authors:** Hanah Muflihanah, Mochammad Hatta, Ente Rood, Pauline Scheelbeek, Theresia H Abdoel, Henk L Smits

**Affiliations:** 1 Veterinary Diseases Investigation Centre, Maros, South Sulawesi, Indonesia; 2 Molecular Biology and Immunology Laboratory, Department of Microbiology, Faculty of Medicine, Hasanuddin University, Makassar, Indonesia; 3 KIT Biomedical Research, Royal Tropical Institute/Koninklijk Instituut voor de Tropen (KIT), Meibergdreef 39, 1105AZ, Amsterdam, The Netherlands

**Keywords:** Brucellosis, Abortion, Genotyping, Reproduction, Fertility, Prevalence, Pen-side diagnostics, Indonesia, Livestock, Productivity, Cattle

## Abstract

**Background:**

Brucellosis is a major cause of infertility and reproductive failure in livestock. While cattle in the Eastern Indonesian archipelago suffers from reproductive problems information on bovine brucellosis in the region is fragmentary. The control of brucellosis requires a major and prolonged effort and confirmation of the infection by isolation with detailed knowledge of the spread of the infection is essential when planning a control program.

**Results:**

Serological investigation of *Brucella* infection in beef cattle tended under extensive farming conditions revealed a high seroprevalence (19.3%; 95% CI, 17–22) in the compliment fixation tests. The results of a rapid and simple field test correlated well with the Rose Bengal test (kappa, 0.917) and indicated an acceptable sensitivity (87.5%) and specificity (98.1%) compared with the complement fixation test. Reproductive failure was reported for 39.0% of the cows with a loss of calves due to abortion or early death amounting to 19.3%. Past reproductive failure did not, however, correlate with seropositivity in the complement fixation test (RP = 1.21; P = 0.847). *B. abortus* biovar 1 was freshly isolated from the hygromas of two cows and together with thirty banked isolates collected since 1990 from different parts of Sulawesi and Timor eight related genotypes could be distinguished with one genotype being identical to that of an isolate (BfR91) from Switzerland. The Indonesian genotypes formed together with BfR91 and one African and one North American isolate a distinct branch on the *B. abortus* biovar 1 dendogram.

**Conclusions:**

Bovine brucellosis appears to be widespread in the Eastern Indonesian archipelago and calls for urgent intervention. The fresh isolation of the pathogen together with the observed high seroprevalence demonstrates the presence and frequent exposure of cattle in the area to the pathogen. The application of a rapid and simple field test for brucellosis could be very useful for the quick screening of cattle at the pen side.

## Background

Brucellosis is a major abortifacient zoonotic agent of livestock with a worldwide occurrence [1]. Bovine brucellosis is caused by infection with *Brucella abortus*[2]. This species and *B. melitensis* and *B. suis,* the two other *Brucella* species of veterinarian importance, are highly infectious and pathogenic organisms that cause infertility, abortion and low productivity in their natural hosts [2]. Establishment of the carrier state in a large proportion of animals can lead to a major reduction in milk yield which together with losses through abortion or early calf death due to *B. abortus* infection is a huge economic constraint for farmers [3,4]. In regions where disease surveillance and control measures are not instigated, long-term chronic infections are often associated with carpal hygromas and infertility [5]. Disease presentations in bulls include orchitis, epididymitis and seminal vesiculitis [6]. The ability of the pathogen to survive and replicate within different host cells explains its pathogenicity. Extensive replication in placental trophoblasts is associated with abortion, and persistence in macrophages and other cell types leads to chronic infections [7]. Chronically infected cattle may shed the organism via milk and reproductive tract discharges, and can also vertically transmit infection to subsequently born calves, thereby maintaining disease transmission. Aborted fetuses from infected animals contain huge numbers of infectious organisms and if not properly disposed form a major source of contamination. The pathogen is highly contagious and is easily spread by licking of infected animals and abortion materials, and abortion materials, discharges and waste of infected animals may contaminate stables, meadows, food supplies and water sources. Direct contact with infected animals and consumption of contaminated dairy may cause infection in human beings [8].

Although few studies have reported the presence of brucellosis in livestock in Indonesia, the infection could well be widespread also because of unrestricted trade between different provinces and islands and the absence of a coherent control policy and surveillance system. In Indonesia, brucellosis was isolated from cattle in Java as early as 1915 [9]. Subsequent serological studies have indicated the presence of bovine brucellosis in cattle in different islands of the Indonesian archipelago including South Sulawesi and West Timor [10,11]. Porcine brucellosis has been reported in pigs in Java with a seroprevalence in the Rose Bengal test (RBT) of 22.3% for pigs slaughtered in Kapuk Jakarta in West Java and of 14.9% for animals tested at a slaughterhouse in Surabaya in East Java [12]. Infection with *B. suis* biovar 1 was confirmed by isolation. Although infection of goats and sheep in Indonesia has not been documented, *B. melitensis* is likely to be present as well. The consumption of milk and other dairy is not popular in Indonesia and possibly for that reason and because of lack of awareness and absence of diagnostic facilities human cases have not been documented in recent decades. However, farmers, veterinarians and butchers constitute potential risk groups and the diagnosis is easily overlooked as symptoms and signs of brucellosis are non-pathognomonic [13]. For the control of bovine brucellosis an effective vaccine is available [14,15].

Rearing beef cattle is an important and often the only source of income for the numerous small farm holders found throughout the Eastern Indonesian archipelago. Most cattle kept in the area is indigenous Bali breed [16]. The small scale extensive farming systems employed mainly include stall-feeding with grasses, crop residues and or agro-industrial by-products, and roadside and communal grazing with animals tethered or allowed access to grassland, stubble fields or forest areas. The frequent contact between herds and the generally poor sanitary conditions at farms likely contribute to the transmission and spread of pathogens. Cattle farmers in east Indonesia cope with low productivity and reproductive failure is common. Good quantitative information on brucellosis in the livestock population is essential for demonstrating the benefits of intervention. The aim of the study was to investigate the presence of bovine brucellosis and role of brucellosis in the high incidence of reproductive failure of cattle in South-Sulawesi. To this end an inventory of reproductive problems was made and the seroprevalence was determined for a random selection of cattle farms and animals. Isolation of the pathogen from hygroma fluid samples was attempted to confirm the presence of *Brucella* and to examine the diversity and spread of *Brucella* strains isolates were characterized by genotyping and results were compared with the genotypes of a collection of *Brucella* strains previously obtained from cattle in various parts of Sulawesi and East Timor, another main island in the Eastern Indonesia archipelago. Serological testing requires a major logistic effort with transport of samples to a central laboratory that may delay reporting and use of test results. Thus, to simplify testing we took advantage of this study to evaluate a simple and rapid field test for the serodiagnosis of bovine brucellosis that may be used at the pen side [17-19].

## Results

### Brucella seroprevalence and reproductive failure

**Figure 1:**
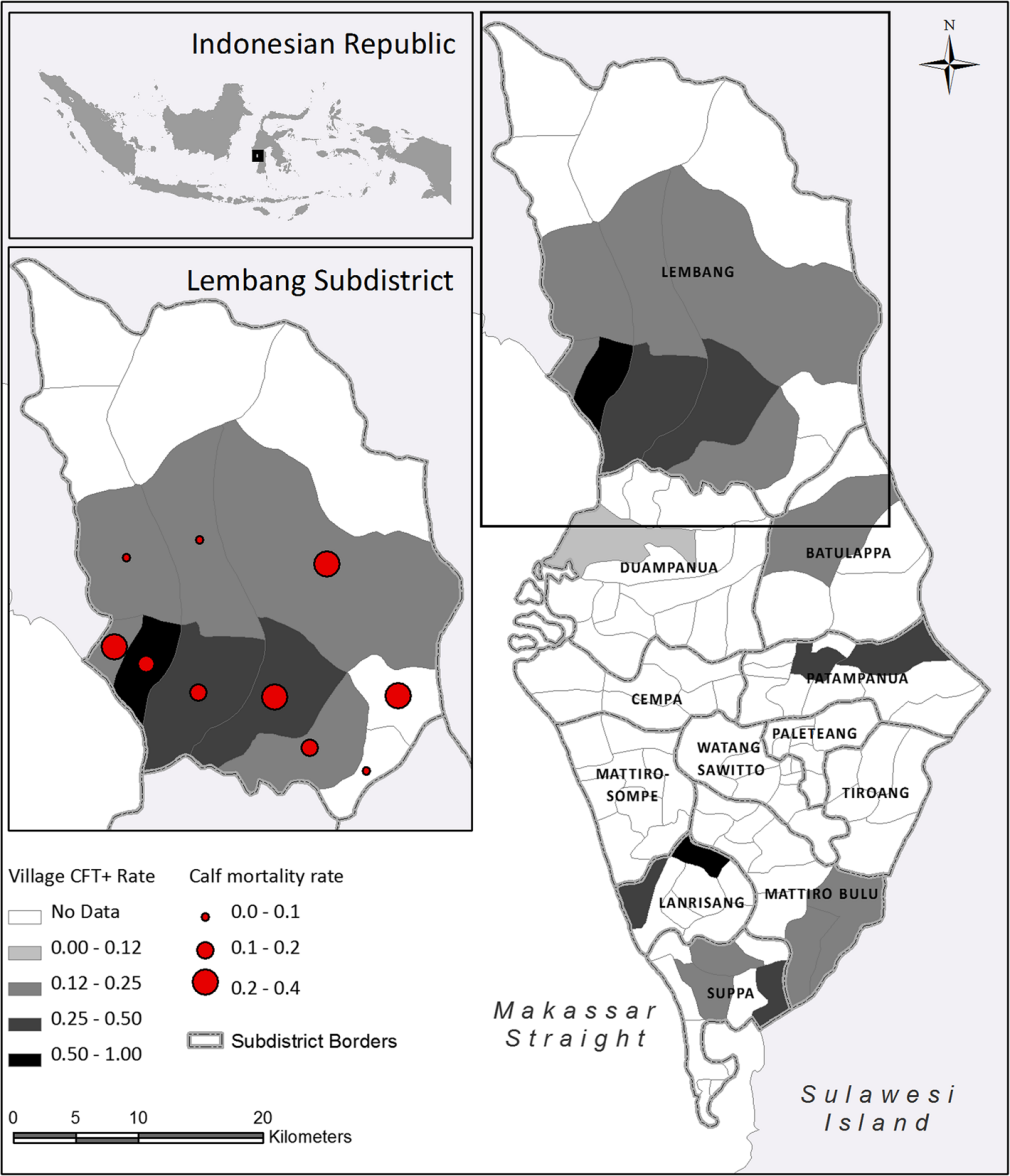
**Distribution of complement fixation test positive cattle in villages in subdistricts of a major cattle rearing district of South Sulawesi and correlation with reproductive problems in the Lembang subdistrict.** Map of Pinrang showing the prevalence rate of CFT seropositive cows in different subdistricts and villages and a comparison of CFT seropositive cows with cows with a history of reproductive (abortion and early death of calf) problems for the Lembang subdistrict (insert). Subdistricts and villages with no data were not samples because cattle was not present or a low number of cattle was present only.

The Pinrang district is one of the major cattle rearing areas in South Sulawesi with a total number of cattle amounting to 43.208 and a cattle density of 22 per km^2^. The average seropositivity for brucellosis in cattle was 18.3% (95% CI, 17–21) in the RBT, 19.3% (95% CI, 17–22) in the CFT, and 21.9% (range, 3.4-50%) for the two assays combined (Table 1). Information on reproductive problems was collected from farmers in the Lembang subdistrict. The cattle density for this subdistrict was 23.7 km^2^ and the seropositivity rate in the CFT was 30.2% (95% CI, 28–33). Of the 182 cows included in this subdistrict 149 (81.9%) had given birth to an average of 2.9 calves (total 534 calves) of which 103 (19.3%) calves from 71 (39.0%) cows aborted or died shortly after birth (Table 2). The percentage of cows with a history of reproductive problems significantly (P < 0.001) increased with age. The average age of the cows with reproductive problems was 6.8 year (range, 3–12) compared with 4.9 year (range, 1–13) for all cows. Seropositivity did not (P = 0.2) increase with age. The prevalence ratio (PR) for abortion and or death of calf was slightly (PR = 1.21; P = 0.249), but not significantly increased for CFT positive cows in comparison with seronegative cows (Table 3). No correlation was observed between current pregnancy and CFT seropositivity (PR = 0.9; P = 0.385). The distribution of CFT test seropositive cows over the different subdistricts in Pinrang and the village of the Lembang subdistrict is presented in Figure 1. The distribution of cows with a history of abortion or a death calf in villages in Lembang did not correlated with the distribution of seropositive cows (insert to Figure 1) and did not show spatial clustering (Morans I = 0.223, P = 0.785).

**Table 1:** Sample size and seroprevalence in cattle in the twelve subdistricts of the Pinrang district, South Sulawesi

						**No. cows positive in the following assay (%)**		
**Subdistrict**	**Cattle population (%)**	**Cattle density (No./km2)**	**No. villages**	**Random sample size**	**Actual sample size**	**RBPT**	**CFT**	**RBPT and or CFT**
Suppa	6.146 (14.2%)	82.7	10	44.8	45	4 (8.8%)	10 (22.2%)	10 (22.2%)
Mattiro Bulu	6.258 (14.5%)	47.2	9	45.6	57	8 (14.3%)	6 (10.5%)	9 (15.8%)
Watang Sawitto	196 (0.5%)	3.3	8	1.4	3	0	0	0
Paleteang	213 (0.5%)	5.7	6	1.6	2	0	0	0
Tiroang	114 (0.2%)	1.8	5	0.8	0	NA	NA	NA
Lanrisang	704 (1.6%)	9.6	7	5.1	4	1 (25.0%)	2 (50%)	2 (50.0%)
Mattiro Sompe	1.722 (4.0%)	17.8	9	12.6	14	1 (7.1%)	3 (21.4%)	3 (8.8%)
Duampanua	4.292 (9.9%)	14.3	14	31.3	35	0	2 (5.7%)	2 (5.7%)
Cempa	581 (1.4%)	6.4	7	4.2	7	0	0	0
Lembang	17.365 (40.2%)	23.7	14	126.6	182	56 (30.8%)	55 (30.2%)	57 (31.3%)
Patampanua	1.970 (4.6%)	14.4	10	14.4	15	1 (7.14%)	2 (14.3%)	2 (13.3%)
Batulappa	3.647 (8.4%)	22.9	5	26.6	29	1 (3.4%)	1 (3.4%)	1 (3.4%)
Total	43.208	22.0	104	315	393	72 (18.3%)	76 (19.3%)	86 (21.9%)

**Table 2:** **Fertility, reproductive failure, *Brucella *** **serostatus and age of cows in the Lembang subdistrict**

**Age group (years)**	**No. cows in age group (%)**	**No. pregnant cows (%)**	**No. cows with a past pregnancy (%)**	**No. deliveries (mean; range)**	**No. abortions and death calves (%)**	**No. cows aborting and or with death calf (%)**	**No. CFT positive cows (%)**
1-4	71 (39.0)	37 (52.1)	48 (67.6)	94 (1.3; 0–6)	18 (19.1)	12 (16.9)	17 (23.9)
5-8	90 (49.5)	47 (52.2)	80 (78.9)	288 (3.2; 0–6)	72 (25.0)	44 (48.9)^1^	29 (32.2)^2^
≥9	21 (11.5)	10 (47.6)	21 (100)	152 (7.2; 4–10)	35 (23.0)	15 (71.4)^1^	9 (42.9)^2^
Total	182 (100%)	94 (51.6)	149 (81.9)	534 (2.9; 0–10)	103 (19.3)	71 (39.0)	55 (30.2)

^1^P < 0.001 and ^2^P = 0.2 for increase with age.

**Table 3:** ***Brucella *** **serostatus, pregnancy and reproductive failure**

**Group**	**CFT negative**	**CFT positive**	**Prevalence ratio**	**P value**
Aborted and or death calf	47	24	1.21	0.249
Control	80	31		
Pregnant	67	27	0.90	0.385
Control	60	28		

### Brucella abortus genotypes in the Eastern Indonesian archipelago

MLVA-16 genotyping of two freshly isolated *Brucella* isolates (BruSS41 and BruSS45) cultured from hygroma fluid collected during the 2011 serosurvey from two seropositive cows present at farms in Pinrang revealed the presence of a *B. abortus* genotype with a close homology (distance = 2) to several *B. abortus* biovar 1 isolates from the United States (strains BCCNV1 and BCCNV5) and Switzerland (strains BfR91 and BfR99) previously described by Le Fleche and coworkers [20] (Table 4). These two Indonesian genotypes are named BInd41 and BInd45. Strains BCCNV1 and BCCNV5 from the US are also known as the vaccine strains S19 and RB51, respectively. The collection of 25 *Brucella* isolates isolated between 1995 and 2011 from cattle in Sulawesi stratified in six additional *B. abortus* biovar 1 MLVA-16 genotypes, named BInd01, BInd03, BInd05, BInd19, BInd33 and BInd37 (Table 4). The MLVA-16 profile of one of these genotypes (BInd33) was identical to that of the previously characterized *B. abortus* biovar 1 strain BfR91 from Switzerland [20]. The two genotypes determined for five *Brucella* isolates from East Timor had also been isolated in Sulawesi (Table 5). The maximum distance between the eight Indonesian genotypes was 5.

**Table 4:** **Determination of species, biovar and genotype of *Brucella *** **isolates by multi-loci variable tandem repeat analysis**

	**Panel 1 loci**								**Panel 2A loci**			**Panel 2B loci**						
**Type isolate (No. of isolates)**	**06**	**08**	**11**	**12**	**42**	**43**	**45**	**55**	**18**	**19**	**21**	**04**	**07**	**09**	**16**	**30**	**Genotype**	**Closest related strain(s), country of isolation (species; distance)**
BruSS01 (19)	4	6	4	12	2	3	3	3	6	21	8	3	4	3	3	6	BInd01	BfR91, Zwitserland (*B. abortus* biovar 1; 2)
BruSS03 (3)	4	6	4	12	2	3	3	3	6	21	8	3	4	3	3	8	BInd03	BfR 91 (*B. abortus* biovar 1; 2)
BruSS05 (2)	4	5	4	12	2	3	3	3	6	21	8	3	4	3	3	6	BInd05	BfR 91 (*B. abortus* biovar 1; 1)
BruSS19 (1)	4	6	4	12	2	3	3	3	6	21	8	3	6	3	3	6	BInd19	BCCNV5 (alias RB51), United States (US) (*B. abortus* biovar 1; 3)
BruSS33 (4)	4	5	4	12	2	3	3	3	6	21	8	3	4	3	3	5	BInd33	BfR 91 (*B. abortus* biovar 1; 0)
BruSS37 (1)	4	5	4	12	2	3	3	3	6	21	8	3	4	3	2	6	BInd37	BfR 91 (*B. abortus* biovar 1; 2)
BruSS41 (1)	4	5	4	12	2	3	3	3	6	21	8	3	5	3	3	5	BInd41	BCCNV1 (alias B19), US/ BCCNV5/BfR91 (*B. abortus* biovar 1; 2)
BruSS45 (1)	4	5	4	12	2	3	3	3	6	21	8	3	5	3	3	6	BInd45	BfR99, Zwitserland/BCCNV1/ BCCNV5/BfR91 (*B. abortus* biovar 1; 2)

**Table 5:** **Origin and genotype of *Brucella abortus *** **biovar 1 isolates from the Eastern Indonesian archipelago**

**No.**	**Isolate**	**Sample type**	**Year**	**Location**	**Genotype**	**District (Province)**
1	BruSS21	Lymphoglandular	1990	Abattoir	BInd01	Makassar (South-Sulawesi)
2	BruSS23	Lymphoglandular	1992	Abattoir	BInd01	Makassar (South-Sulawesi)
3	BruSS20	Lymphoglandular	1994	Abattoir	BInd01	Makassar (South-Sulawesi)
4	BruSS22	Lymphoglandular	1994	Abattoir	BInd01	Makassar (South-Sulawesi)
5	BruSS24	Lymphoglandular	1994	Abattoir	BInd01	Makassar (South-Sulawesi)
6	BruSS19	Hygroma	1997	Farm	BInd19	Makassar (South-Sulawesi)
7	BruSS06	Lymphoglandular	1998	Abattoir	BInd01	Makassar (South-Sulawesi)
8	BruSS09	Lymphoglandular	1998	Abattoir	BInd01	Makassar (South-Sulawesi)
9	BruSS10	Lymphoglandular	1998	Abattoir	BInd01	Makassar (South-Sulawesi)
10	BruSS11	Lymphoglandular	1998	Abattoir	BInd01	Makassar (South-Sulawesi)
11	BruSS12	Lymphoglandular	1998	Abattoir	BInd01	Makassar (South-Sulawesi)
12	BruSS13	Lymphoglandular	1998	Abattoir	BInd01	Makassar (South-Sulawesi)
13	BruSS14	Lymphoglandular	1998	Abattoir	BInd01	Makassar (South-Sulawesi)
14	BruSS07	Lymphoglandular	1998	Abattoir	BInd03	Makassar (South-Sulawesi)
15	BruSS08	Lymphoglandular	1998	Abattoir	BInd03	Makassar (South-Sulawesi)
16	BruSS05	Lymphoglandular	1998	Abattoir	BInd05	Makassar (South-Sulawesi)
17	BruSS01	Hygroma	1995	Farm	BInd01	Wajo (South-Sulawesi)
18	BruSS17	Hygroma	1997	Farm	BInd01	Kendari (South East-Sulawesi)
19	BruSS32	Hygroma	2011	Farm	BInd05	Bone (South-Sulawesi)
20	BruSS37	Hygroma	2011	Farm	BInd37	Bone (South-Sulawesi)
21	BruSS33	Hygroma	2011	Farm	BInd33*	Bone (South-Sulawesi)
22	BruSS34	Hygroma	2011	Farm	BInd33	Bone (South-Sulawesi)
23	BruSS35	Hygroma	2011	Farm	BInd33	Bone (South-Sulawesi)
24	BruSS36	Hygroma	2011	Farm	BInd33	Bone (South-Sulawesi)
25	BruSS30	Hygroma	2009	Farm	BInd01	Pinrang (South-Sulawesi)
26	BruSS41	Hygroma	2011	Farm	BInd41	Pinrang (South-Sulawesi)
27	BruSS45	Hygroma	2011	Farm	BInd45	Pinrang (South-Sulawesi)
28	BruSS16	Hygroma	1997	Farm	BInd01	East Timor
29	BruSS15	Hygroma	1998	Farm	BInd01	East Timor
30	BruSS02	Hygroma	1998	Farm	BInd01	East Timor
31	BruSS18	Hygroma	1998	Farm	BInd01	East Timor
32	BruSS03	Hygroma	1998	Farm	BInd03	East Timor

*Genotype identical to the genotype previously characterized for an isolate (BfR91) obtained in 1998 from a cow in Switzerland [20].

**Figure 2:**
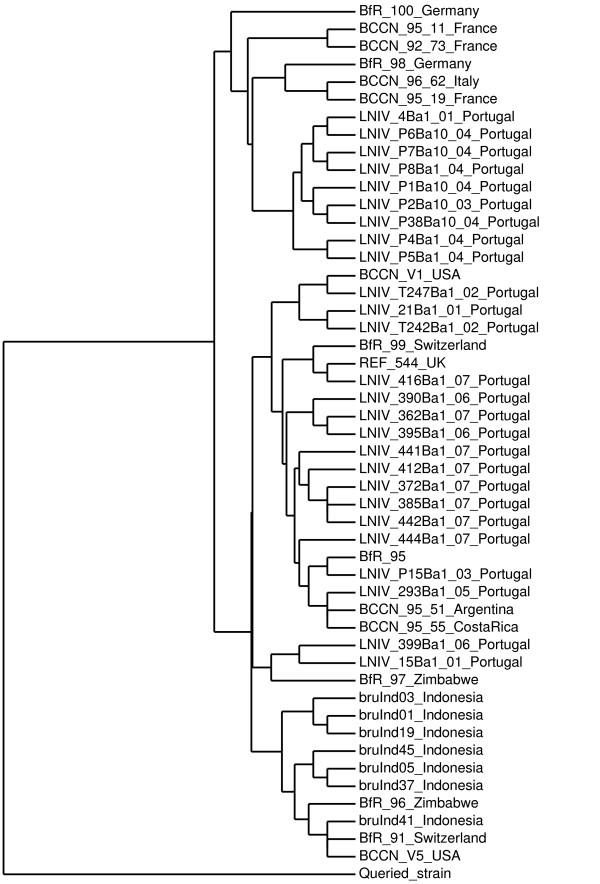
**Dendogram of global *Brucella abortus *biovar 1 genotypes.** Dendogram based on MLVA-16 genotyping showing the relationship of 49 *B. abortus* biovar 1 strains originating from various continents including Europe, South, Central and North America, Africa and Asia whereby the Indonesian genotype BInd33 is identical to genotype BfR91 from Switzerland. The dendogram was constructed using *B. melitensis* biovar 1 strain BCCNV3 with Bruce MLVA-16 code (3, 4, 2, 13, 4, 2, 3, 3, 7, 18, 6, 2, 5, 6, 8, 4) as queried strain [39].

Forty-two MLVA-16 genotypes have been reported for *B. abortus* biovar 1 isolates from the old and new world and Africa combined [20,21], which together with the eight genotypes determined for the Indonesia isolates makes 49 distinct *B. abortus* biovar 1 genotypes. The different MLVA-16 panel 1 and 2A loci for this global collection of *B. abortus* biovar 1 genotypes showed limited variation with 1, 2 or 3 alleles only and a modest variation was observed for four of the panel 2B locus with a maximum *D* value of 0.729 and with 6 alleles observed for MLVA-16 locus bruce16 (Table 6). The maximum distance between the genotypes of this collection of 44 *B. abortus* biovar 1 genotypes was ten. In the dendogram constructed for this global collection of *B. abortus* biovar 1 genotypes from Europe, Africa and the Americans, the Indonesian isolates locate on a branch together with three genotypes originally detected in Europe (BfR91), Africa (BfR96) and North America (BCCNV5) (Figure 2).

**Table 6:** **Hunter-Gaston diversity index for MLVA-16 loci of the global collection of *Brucella abortus *** **biovar 1 genotypes from Europe, North, Central and South America, Africa and Indonesia**

**Locus (panel)**	**No. of alleles**	**No. of repeats**	**D value (95% confidence interval)**
Bruce06 (1)	2	3, 4	0.327 (0.193-0.460)
Bruce08 (1)	2	5, 6	0.115 (0.000-0.231)
Bruce11 (1)	1	4	0.000 (0.000-0.132)
Bruce12 (1)	2	12, 13	0.040 (0.000-0.115)
Bruce42 (1)	2	1, 2	0.444 (0.351-0.537)
Bruce43 (1)	2	2, 3	0.350 (0.222-0.479)
Bruce45 (1)	1	3	0.000 (0.000-0.132)
Bruce55 (1)	2	1, 3	0.040 (0.000-0.115)
Bruce18 (2A)	3	4, 5, 6	0.079 (0.000-0.181)
Bruce19 (2A)	1	42	0.115 (0.000-0.231)
Bruce21 (2A)	2	6, 8	0.040 (0.000-0.115)
Bruce04 (2B)	3	3, 4, 5	0.496 (0.362-0.629)
Bruce07 (2B)	4	4, 5, 6, 7	0.433 (0.279-0.588)
Bruce09 (2B)	1	3	0.000 (0.000-0.132)
Bruce16 (2B)	6	0, 2, 3, 4, 6, 8	0.729 (0.654-0.804)
Bruce30 (2B)	5	0, 4, 5, 6, 8	0.707 (0.654-0.760)

### Diagnostic characteristics of a rapid and simple pen-side diagnostic for bovine brucellosis

The study was used to confirm the diagnostic performance of a rapid and simple field assay for the serodiagnosis of brucellosis in cattle [17]. Application of the field test on all 393 samples included in this study resulted in a seropositivity of 19.3% (95% CI, 17–21) and based on the results of the CFT the sensitivity and specificity of this field test was 87.5% (95% CI, 81–92) and 98.1% (95% CI, 96–99), respectively, compared with 83.8% (95% CI, 77–88) and 98.4% (95% CI, 97–99) for the RBPT. Four of the 10 CFT positive samples that failed to react in the LFA had CFT titers of 1:4, three a titer of 1:8 and three a titer of 1:16. Results of the LFA and RBPT showed a high level of agreement (kappa value, 0.917).

## Discussion

The observed very high seroprevalence (19.3% in CFT) of brucellosis in cattle in the Pinrang district of South Sulawesi in the Eastern Indonesian archipelago and the recent isolation of the pathogen from two seropositive cows clearly demonstrated the urgent need for the instigation of appropriate control measures based on mass vaccination. The presence of brucellosis in livestock is detrimental to the production system as it causes abortion, weak siblings and reduced fertility. Information collected from farmers in one subdistrict of Pinrang confirmed that their animals suffered from high abortion rates (11.4%) and mortality during or shortly after birth (7.9%) with 39.0% of the cows having had reproductive problems. With increasing age reproductive problems markedly increased and 71.4% of animals in the higher age group (≥9 year) had suffered from one or more reproductive failure. Various reports from the African continent have demonstrated an association between seropositivity and present or past abortion in livestock [22-30]. However, no correlation between CFT seropositivity for brucellosis and reproductive problems was found in this study. Cattle with reproductive failure did not show spatial clustering and areas with high reproductive failure did not correlate with high CFT positivity. A possible explanation is that current seropositivity in the CFT test does not reflect a high rate of active circulation of the pathogen and that some seropositive animals were recently exposed to the pathogen without fully supporting infection. Abortion in cattle is a manifestation of acute disease and current seropositivity may not reflect the occurrence of a past infection that caused reproductive failure. Serological testing may not be sensitive enough to detect residual antibody levels of an infection that caused reproductive failure in the past and that has been resolved by the immune system. Also, reproductive failure could be the result of infection by other abortifacient pathogens such as bovine viral disease, leptospirosis, *Toxoplasma gondii*, *Neospora caninum, Campylobacter ssp.,* and *Ornithodoros coriaceus*, and further studies are needed to investigate the presence of these agents [31-34]. A recent study investigating reproductive failure in cattle in Ethiopia indicated that *Neospora caninum* infection might have a greater impact than infection with *Brucella*[35]. Moreover, the risk of abortion could be increased for co-infections [36]. Nevertheless, bovine brucellosis appears to be widespread in the Eastern Indonesian archipelago: a summary of our laboratory records for samples submitted during the past two year for routine serological testing for brucellosis revealed that seropositive animals have been detected in thirteen out of 30 districts in Sulawesi (N = 2.429 samples; 14.6% seropositive, range 0-100%) investigated, in four out of five districts in the Maluku (N = 768 samples; 3.4% seropositive, range 0–14.4%), and in one out of five districts in Papua (N = 80 samples; 2.5% seropositive, range 0–33.3%). Transmission and spread of *B. abortus* is by intrauteral infection of the fetus, through ingestion of contaminated milk by offspring and through direct or indirect mucosal contact with fluids and tissue associated with birth or abortion of infected fetuses [37]. If farm sanitation is insufficient and infected animals are not kept separated, stables, meadows, food stocks and water points may all become contaminated and function as sources for further transmission. Several factors that perpetuate the transmission of infectious diseases are present in South Sulawesi. Sanitary conditions are poor at many farms, cattle are brought to common water sources for drinking and may be kept at common pastures during the day. The relatively high cattle density is another factor of concern [2]. In addition, knowledge of farmers of infectious agents and of preventive measures is very limited. Discussion with farmers in the study area have indicated that most farmers consider abortion as a natural but premature delivery and do not associate abortion with disease. Similarly, farmers do not recognize hygroma as a disease presentation. Migration and trade of livestock and the absence of control measures could be important as well. Clearly better information to inform farmers about the causes, consequences and risks of infection is needed [38]. Given the high seroprevalence a well-designed disease education and information program for farmers could be an essential components of a brucellosis control and prevention program. Awareness of farmers of the risks and consequences of infection will increase commitment to participate and contribute to the success of such a program by accepting and implementing measures.

The RBT as screening test together with the CFT for confirmation is considered adequate for the serodiagnosis of *Brucella* infection in livestock. Direct proof for the infection is obtained after isolation of the pathogen by culture. During the field work two cows were identified with an hygroma at the knee. Hygroma fluid was collected from both cows and after culture isolates were obtained and identified as *B. abortus* biovar 1 by classical biotyping*.* The two cows turned out to be seropositive. Because of practical and logistic reasons no attempts were made to culture *Brucella* from a larger series of seropositive animals identified during the field work. However, MLVA-16 genotyping of a collection of *Brucella* isolates cultured from hygroma fluids and lymphogranular biopsies established between 1990 and 2011 from cows in various districts in South Sulawesi revealed the presence of a confined group of six *B. abortus* biovar 1 genotypes that appeared to be very closely related to the two genotypes identified during the present serosurvey. One of the genotypes (BInd33) appeared to be identical to that of a *B. abortus* biovar 1 strain (strain BfR91) isolated in 1996 from a cow in Switzerland [20]. Two *B. abortus* biovar 1 genotypes found in South Sulawesi had also been isolated from cattle in East Timor indicating their widespread distribution in the archipelago. Cattle is frequently traded between the various islands of the Indonesian archipelago and without testing and the enforcement of transport restrictions for positive livestock pathogens may be easily spread to other islands and provinces.

In the *B. abortus* biovar 1 dendogram the Indonesian isolates seem to form a distinct branch that also encompass the BfR91 isolate from Switzerland that is identical to one of the Indonesian genotypes (BInd33), a genotype identified in Zimbabwe (genotype BfR96) and the RB51 vaccine strain (genotype BCCNV5) that was isolated in the USA. The dendogram contains two other main branches made up by isolates mainly from Portugal. It should be noted that genotyping has been done for isolates from only few countries and that the number of isolates originating from Africa, the Americas and Asia that have been characterized is still very small making the picture of the variation and geographic distribution of *B. abortus* biovar 1 genotypes incomplete. The close genetic relationship of the Indonesian genotypes with specific genotypes from Europe, Africa and America could indicate that pathogens with very similar genotype have been spread to countries on different continents. In the past cattle such as Frisian breed was imported in Indonesia from other continents including Europe and Australia. Alternatively, genotypes may have been evolved independently in different geographic regions. The low degree of diversity in MLVA-16 pattern for the *B. abortus* isolates from Indonesia implies that the value of MLVA-16 genotyping for source tracing as suggested in a previous study is limited [39]. The BInd01 genotype was isolated nineteen times during a period of almost two decades and from cattle examined at widely different geographic locations in South Sulawesi, Southeast Sulawesi and East Timor demonstrating the enormous risk of spreading the infection if measures to control transmission are not in place and enforced.

The CFT and the RBT are complex and time consuming and require a dedicated laboratory. The *Brucella* LFA is very simple to perform and easily can be used in the field. Consistent with earlier observations this assay is highly specific and sensitive and can be used as a user-friendly field test for the rapid assessment of infection with *Brucella*. The estimated sensitivity of 87.5% and specificity of 98.1% calculated for the LFA are well in agreement with earlier reports of studies performed in Portugal [8], Cameroon [9] and Nigeria [10]. The field teams considered the assay to be fairly easy and rapid to perform in the field. While the *Brucella* field assay may be used to access the presence and importance of bovine brucellosis in an area additional screening in CFT may be needed to exclude false-negative results. Notably, only few samples all with low CFT titers tested false-negative in the rapid test. Additional testing in the CFT would be useful if a control policy in addition to vaccination would include segregation of infected animals or a test and slaughter strategy.

## Conclusions

*B. abortus* biovar 1 was isolated beef cattle in a major cattle rearing area with reproductive problems in South Sulawesi. *B. abortus* biovar 1 isolates from the Eastern Indonesian archipelago consisted of a confined group of closely related genotypes with one genotype identical to an isolate from Switzerland. A high seroprevalence was measured which however did not correlate with a history of reproductive failure. The potential detrimental effect of brucellosis on the productivity of the livestock sector calls for an urgent need for the development of a coherent control policy.

## Methods

The research protocol for this study was approved by the ethical committee of the Hasanuddin University.

### Study area, sample size and data collection

Because of the large number of cattle samples submitted for laboratory investigation from several districts of South Sulawesi testing positive for brucellosis and the growing concern about the health and productive of the cattle population, the central Indonesian government allocated a budget for vaccination of cattle in high prevalence areas. In preparation of the vaccination campaign this seroprevalence study for bovine brucellosis was executed in the Pinrang district. This district is one of twenty-three districts of the South Sulawesi province and encompasses one of the major cattle rearing areas of the province. Previous vaccination for brucellosis in the area took place more than a decade ago. The planned vaccination campaign will use the S19 vaccine [14,15]. The Pinrang district is divided in 12 subdistricts with 104 villages and 320.000 inhabitants and has a cattle population of 43.208 cows. Farmers in the district are registered and are obliged to keep written records documenting their livestock. These records include information on number and age of livestock present at the farm, reproduction, health issues and vaccination. Based on the livestock census data for the different subdistricts and villages and an assumed seroprevalence of 7%, a desired 95% agreement level, an accepted 5% error, a sample size calculation was performed using the Win Episcope 2.0 software package. The total samples size was calculated to be 315 (0.73%) cows at the district level with 5 to 14 cows per subdistrict and zero to 18 cows per village in dependence of the number of animals present. Farms and cows to be tested were selected by drawing random numbers. The cattle population and the number of cows tested in each subdistrict is indicated in Table 1. Subdistricts with a small cattle population resulting in a sample size <1 were not included in the study. Different field teams visited the different subdistrict and information on reproduction and reproductive problems could be collected from the farmers in Lembang, the subdistrict with the largest cattle population but due to logistic reasons and time constraints this information was not obtained in the other subdistricts. Blood samples were collected from the jugular vein, allowed to cloth and after removal of the blood cloth transported on ice to the laboratory where they were frozen and tested within 1 to 14 days. The samples were collected between June and December 2011. None of the cows included had ever been vaccinated for brucellosis.

### Rose Bengal test

The RBT using antigen was used as screening test for brucellosis [40]. The test was performed in WHO haemagglutination plates by mixing, using a clean rod, one drop antigen (Pusvetma, Surabaya, Indonesia) with one drop serum and incubation on a rotary shaker for exactly 4 minutes after which the test result was read. Any visible reaction was considered to be positive.

### Complement fixation test

The complement fixation test (CFT) was used as confirmatory test [40]. The test was carried out in round bottomed polystyrene microtiter plates using a 2-fold serial dilution of 25 μl heat inactive serum sample mixed with 25 μl antigen (Synbiotics, USA) and 25 μl complement (BBVet, Maros, Indonesia). Plates were incubated at 37°C for 30 minutes after which 25 μl hemolysin sensitized red blood cells was added. After mixing and two further incubation steps first for 30 minutes at 37°C and next for 2–3 hours at 40°C results were read by scoring the degree of hemolysis.

### Brucella lateral flow assay

The *Brucella* lateral flow assay (LFA) device for the serodiagnosis of bovine brucellosis was performed as described previously [17]. Briefly, 10 μl whole blood was applied to the sample well of the assay device immediately followed by the addition of 120 μl running fluid. The assay result was read after 10 to 15 min by visual inspection of the test and control lines for the presence of staining. Samples were scored positive when both the test and control lines stained and negative when staining at control line was observed and the test line remained negative. The LFA was applied on all 393 samples collected during the field work.

### *Brucella* isolates

During the field work for this study seropositive cows with a hygroma at the knee were identified at two farms. Fluid collected from the two hygromas was placed in culture and yielded two *Brucella* isolates (BruSS41 and BruSS45). An additional 30 banked *Brucella* isolates were available for genotyping. These isolates that had been cultured from hygroma fluid and lymphoglandular nodules collected between 1990 and 2011 from cattle at farms and abattoirs sampled in the provinces of South Sulawesi (N = 24), Southeast Sulawesi (N = 1) and East Timor (N = 5). All isolations were done at the Disease Investigation Centre in Maros, South Sulawesi.

### Multiple locus variable number tandem repeat analysis of Brucella genotypes

PCR based on multiple locus variable number tandem repeat analysis (MLVA) genotyping of *Brucella* isolates was performed with MLVA-16 panel 1 (bruce06, -08, -11, -12, -42, -43, -45 and -55) primer sets for species identification and MLVA-16 panels 2A (bruce18, -19 and -21) and 2B (bruce04, -07, -09, -16 and -30) primer sets for further subspecies differentiation [20]. PCR products were separated by electrophoresis on 2% (panel 1) or 3% (panel 2) agarose gels stained with ethidium bromide and viewed by UV illumination. The length of the PCR product was deduced in dependence of the expected tandem repeat unit by comparison with a 100 bp or a 20 bp molecular marker ladder. For each run, DNA control from two reference strains was carried along. In this study, we define a genotype as an isolate with a distinct MLVA-16 pattern. The distance between two genotypes is defined as the minimum number of changes in the number of repeats of any locus that converts one genotype to the other. The Hunter-Gaston index of diversity (*D* value) with 95% Confidence Intervals was calculated using an online tool (http://www.hpa-bioinformatics.org.uk/cgi-bin/DICI/DICI.pl). MLVA-16 patterns were compared with isolates in the public database *Brucella* 2010 (http://mlva.u-psud.fr; accessed May 2012) using cluster analysis performed by unweighted pair group method with arithmetic mean (UPGMA) algorithm [41-43]. Treedyn was used to generate a rooted tree for *B. abortus* biovar 1 MLVA-16 genotypes.

### Statistics

Univariate analysis was used to determine risk factors for being seropositive. Prevalence ratios were calculated using unconditional maximum likelihood estimation and Fisher exact P-values. The agreement beyond chance (kappa value) was calculated to determine the relation between serological tests results obtained with two different tests. Spatial clustering of calf mortality was tested for using Moran’s I statistic.

## Competing interests

The authors declare that they have no competing interests.

## Authors’ contributions

HM, HLS and MH initiated and designed the study, HLS drafted the manuscript, M supervised the field activities and the microbiology and serology work, THA performed the genetic typing, ER and PS performed the statistical analysis. All authors have read and approved the manuscript.

## References

[B1] Pappas G Papadimitriou P Akritidis N Christou L Tsianos EV The new global map of human brucellosis Lancet Infect Dis 2006 6 91 99 10.1016/S1473-3099(06)70382-6 16439329

[B2] Schmidt MK Muslimatun S West CE Schultink W Gross R Hautvast JGAJ Nutritional status and linear growth of indonesian infants in west java are determined more by prenatal environment than by postnatal factors J Nutr 2002 132 2202 2207 12163663 10.1093/jn/132.8.2202

[B3] Zinsstag J Schelling E Roth F Bonfoh B de Savigny D Tanner M Human benefits of animal interventions for zoonosis control Emerg Infect Dis 2007 13 527 531 10.3201/eid1304.060381 17553265 PMC2725951

[B4] Aiello SE, Mays A The Merck Veterinary Manual 1998 8 Whitehouse station, NJ, USA: Merck & CO., Inc

[B5] McDermott JJ Arimi SM Brucellosis in sub-Saharan Africa: epidemiology, control and impact Vet Microbiol 2002 90 111 134 10.1016/S0378-1135(02)00249-3 12414138

[B6] Carvalho Neta AV Mol JP Xavier MN Paixão TA Lage AP Santos RL Pathogenesis of bovine brucellosis Vet J 2010 184 146 155 10.1016/j.tvjl.2009.04.010 19733101

[B7] Godfroid J Scholz HC Barbier T Nicolas C Wattiau P Fretin D Whatmore AM Cloeckaert A Blasco JM Moriyon I Saegerman C Muma JB Al Dahouk S Neubauer H Letesson JJ Brucellosis at the animal/ecosystem/human interface at the beginning of the 21st century Prev Vet Med 2011 102 118 131 10.1016/j.prevetmed.2011.04.007 21571380

[B8] Franco MP Mulder M Gilman RH Smits HL Human brucellosis Lancet Infect Dis 2007 7 775 786 10.1016/S1473-3099(07)70286-4 18045560

[B9] Danusnatoso H Joseph SW Sidarta H A review of brucellosis in Indonesia with a report of a recent case Southeast Asian J Trop Med Public Health 1972 3 314 318 4631508

[B10] Makka D Hutabarat TSPN Sudana IG Abdul Madjid M Kanyon SJ Epidemiology of brucellosis in smallholder cattle herds in South-Sulawesi, Indonesia. Proc. 5^th^ International Symposium Vet. Epidemiology and Economics Acta Vet Scand 1988 Suppl 84 240

[B11] Geong M Robertson ID Response of Bali cattle (Bos javanicus) to vaccination with Brucella abortus strain 19 in West Timor Prev Vet Med 2000 47 177 186 10.1016/S0167-5877(00)00174-4 11058778

[B12] van der Giessen JW Priadi A Swine brucellosis in Indonesia Vet Q 1988 10 172 176 10.1080/01652176.1988.9694167 3176296

[B13] Dean AS Crump L Greter H Hattendorf J Schelling E Zinsstag J Clinical manifestations of human brucellosis: a systematic review and meta-analysis PLoS Negl Trop Dis 2012 6 e1929 10.1371/journal.pntd.0001929 23236528 PMC3516581

[B14] Moriyón I Grilló MJ Monreal D González D Marín C López-Goñi I Mainar-Jaime RC Moreno E Blasco JM Rough vaccines in animal brucellosis: structural and genetic basis and present status Vet Res 2004 35 1 38 10.1051/vetres:2003037 15099501

[B15] Mukherjee F Jain J Grilló MJ Blasco JM Nair M Evaluation of Brucella abortus S19 vaccine strains by bacteriological tests, molecular analysis of ery loci and virulence in BALB/c mice Biologicals 2005 33 153 160 10.1016/j.biologicals.2005.04.003 16081301

[B16] Purwantara B Noor RR Andersson G Rodriguez-Martinez H Banteng and Bali Cattle in Indonesia: status and forecasts Reprod Domest Anim 2012 47 s1 2 6 22212203 10.1111/j.1439-0531.2011.01956.x

[B17] Abdoel T Dias IT Cardoso R Smits HL Simple and rapid field tests for brucellosis in livestock Vet Microbiol 2008 130 312 319 10.1016/j.vetmic.2008.01.009 18321664

[B18] Bronsvoort BM Koterwas B Land F Handel IG Tucker J Morgan KL Tanya VN Abdoel TH Smits HL Comparison of a flow assay for brucellosis antibodies with the reference cELISA test in West African Bos indicus PLoS One 2009 4 e5221 10.1371/journal.pone.0005221 19381332 PMC2667634

[B19] Bertu WJ Gusi AM Hassan M Mwankon E Ocholi RA Ior DD Husseini BA Ibrahim G Abdoel TH Smits HL Serological evidence for brucellosis in Bos indicus in Nigeria Trop Anim Health Prod 2012 44 253 258 10.1007/s11250-011-0011-2 22086409

[B20] Le Flèche P Jacques I Grayon M Al Dahouk S Bouchon P Denoeud F Nöckler K Neubauer H Guilloteau LA Vergnaud G Evaluation and selection of tandem repeat loci for a *Brucella* MLVA typing assay BMC Microbiol 2006 6 9 10.1186/1471-2180-6-9 16469109 PMC1513380

[B21] Ferreira AC Chambel L Tenreiro T Cardoso R Flor L Dias IT Pacheco T Garin-Bastuji B Le Flèche P Vergnaud G Tenreiro R de Sá MI MLVA16 typing of Portuguese Human and Animal Brucella melitensis and Brucella abortus isolates PLoS One 2012 7 e42514 10.1371/journal.pone.0042514 22905141 PMC3419166

[B22] Megersa B Biffa D Abunna F Regassa A Godfroid J Skjerve E Seroprevalence of brucellosis and its contribution to abortion in cattle, camel, and goat kept under pastoral management in Borana, Ethiopia Trop Anim Health Prod 2011 43 651 656 10.1007/s11250-010-9748-2 21088890

[B23] Matope G Bhebhe E Muma JB Oloya J Madekurozwa RL Lund A Skjerve E Seroprevalence of brucellosis and its associated risk factors in cattle from smallholder dairy farms in Zimbabwe Trop Anim Health Prod 2011 43 975 982 10.1007/s11250-011-9794-4 21327714

[B24] Muma JB Godfroid J Samui KL Skjerve E The role of Brucella infection in abortions among traditional cattle reared in proximity to wildlife on the Kafue flats of Zambia Rev Sci Tech 2007 26 721 730 18293620

[B25] Haileselassie M Kalayou S Kyule M Asfaha M Belihu K Effect of Brucella infection on reproduction conditions of female breeding cattle and its public health significance in Western Tigray, northern Ethiopia Vet Med Inst 2011 354943 1 7 10.4061/2011/354943 PMC3142704 21822466

[B26] Makita K Fèvre EM Waiswa C Eisler MC Thrusfield M Welburn SC Herd prevalence of bovine brucellosis and analysis of risk factors in cattle in urban and peri-urban areas of the Kampala economic zone, Uganda BMC Vet Res 2011 7 60 10.1186/1746-6148-7-60 22004574 PMC3212899

[B27] Muma JB Pandey GS Munyeme M Mumba C Mkandawire E Chimana HM Brucellosis among smallholder cattle farmers in Zambia: public health significance Trop Anim Health Prod 2012 44 915 920 10.1007/s11250-011-9987-x 21947888

[B28] Gomo C de Garine-Wichatitsky M Caron A Pfukenyi DM Survey of brucellosis at the wildlife-livestock interface on the Zimbabwean side of the Great Limpopo Transfrontier Conservation Area Trop Anim Health Prod 2012 44 77 85 10.1007/s11250-011-9890-5 21643664

[B29] Haileselassie M Kalayou S Kyule M Asfaha M Belihu K Effect of Brucella infection on reproduction conditions of female breeding cattle and its public health significance in Western Tigray, northern Ethiopia Vet Med Inst 2011 2011 354943 10.4061/2011/354943 PMC3142704 21822466

[B30] Tesfaye G Tsegaye W Chanie M Abinet F Seroprevalence and associated risk factors of bovine brucellosis in Addis Ababa dairy farms Trop Anim Health Prod 2011 43 1001 1005 10.1007/s11250-011-9798-0 21331496

[B31] Jimenez DF Perez AM Carpenter TE Martinez A Factors associated with infection by Campylobacter fetus in beef herds in the Province of Buenos Aires, Argentina Prev Vet Med 2011 101 157 162 10.1016/j.prevetmed.2011.05.014 21737166

[B32] Teglas MB Mapes S Hodzic E Nieto NC Co-infection of Ornithodoros coriaceus with the relapsing fever spirochete, Borrelia coriaceae, and the agent of epizootic bovine abortion Med Vet Entomol 2011 5 337 343 21410735 10.1111/j.1365-2915.2011.00952.x

[B33] Shabbir MZ Nazir MM Maqbool A Lateef M Shabbir MA Ahmad A Rabbani M Yaqub T Sohail MU Ijaz M Seroprevalence of Neospora caninum and Brucella abortus in dairy cattle herds with high abortion rates J Parasitol 2011 97 740 742 10.1645/GE-2734.1 21506829

[B34] Yildiz K Kul O Babur C Kilic S Gazyagci AN Celebi B Gurcan IS Seroprevalence of Neospora caninum in dairy cattle ranches with high abortion rate: special emphasis to serologic co-existence with Toxoplasma gondii, Brucella abortus and Listeria monocytogenes Vet Parasitol 2009 164 306 310 10.1016/j.vetpar.2009.06.004 19592171

[B35] Asmare K Regassa F Robertson LJ Martin AD Skjerve E Reproductive disorders in relation to Neospora caninum, Brucella spp. and bovine viral diarrhoea virus serostatus in breeding and dairy farms of central and southern Ethiopia Epidemiol Infect 2013 141 1772 1780 10.1017/S0950268812002191 23034138 PMC9151610

[B36] Escamilla HP Martínez MJ Medina CM Morales SE Frequency and causes of infectious abortion in a dairy herd in Queretaro, Mexico Can J Vet Res 2007 71 314 317 17955907 PMC1940280

[B37] Olsen S Tatum F Bovine brucellosis Vet Clin North Am Food Anim Pract 2010 26 15 27 10.1016/j.cvfa.2009.10.006 20117540

[B38] Smits HL Brucellosis in pastoral and confined livestock: prevention and vaccination Rev Sci Tech 2013 32 219 228 23837379 10.20506/rst.32.1.2200

[B39] Al Dahouk S Flèche PL Nöckler K Jacques I Grayon M Scholz HC Tomaso H Vergnaud G Neubauer H Evaluation of Brucella MLVA typing for human brucellosis J Microbiol Methods 2007 69 137 145 10.1016/j.mimet.2006.12.015 17261338

[B40] Bovine brucellosis Bovine brucellosis Manual of Diagnostic Tests and Vaccines for Terrestrial Animals 2012. Office International de Epizoteshttp://www.oie.int/international-standard-setting/terrestrial-manual/access-online

[B41] Dereeper A Audic S Claverie JM Blanc G BLAST-EXPLORER helps you building datasets for phylogenetic analysis BMC Evol Biol 2010 10 8 10.1186/1471-2148-10-8 20067610 PMC2821324

[B42] Dereeper A Guignon V Blanc G Audic S Buffet S Chevenet F Dufayard JF Guindon S Lefort V Lescot M Claverie JM Gascuel O Phylogeny.fr: robust phylogenetic analysis for the non-specialist Nucleic Acids Res 2008 36 W465 W469 10.1093/nar/gkn180 18424797 PMC2447785

[B43] Chevenet F Brun C Banuls AL Jacq B Chisten R TreeDyn: towards dynamic graphics and annotations for analyses of trees BMC Bioinformatics 2006 7 439 10.1186/1471-2105-7-439 17032440 PMC1615880

